# Prediction of resting energy expenditure in Italian older adults with severe obesity

**DOI:** 10.3389/fendo.2023.1283155

**Published:** 2023-11-08

**Authors:** Ana Lúcia Danielewicz, Stefano Lazzer, Alice Marra, Laura Abbruzzese, Mattia D’Alleva, Maria De Martino, Miriam Isola, Núbia Carelli Pereira Avelar, Vanessa Amaral Mendonça, Ana Cristina Rodrigues Lacerda, Alessandro Sartorio

**Affiliations:** ^1^ Istituto Auxologico Italiano, Istituto di Ricovero e Cura a Carattere Scientifico (IRCCS), Experimental Laboratory for Auxo-endocrinological Research, Piancavallo-Verbania, Italy; ^2^ Department of Health Sciences, Graduate Program in Rehabilitation Sciences, Federal University of Santa Catarina, Araranguá, Santa Catarina, Brazil; ^3^ Department of Medicine, University of Udine, Udine, Italy; ^4^ School of Sport Science, University of Udine, Udine, Italy; ^5^ Istituto Auxologico Italiano, Istituto di Ricovero e Cura a Carattere Scientifico (IRCCS), Division of Eating and Nutrition Disorders, Piancavallo-Verbania, Italy; ^6^ Department of Physiotherapy, Federal University of the Jequitinhonha and Mucuri Valleys, Diamantina, Minas Gerais, Brazil

**Keywords:** energy expenditure, predictive equations, indirect calorimetry, obesity, older adults, aging

## Abstract

**Background:**

In the last decade a large number of studies proposed and/or validated equations to estimate the Resting Energy Expenditure (REE) in adults and/or older adults, however, no equation currently available showed good accuracy for older adults with severe obesity. Thus, this study aimed to develop and validate new predictive equations for REE, based on data from the indirect calorimetry, in Italian older adults with severe obesity.

**Methods:**

A retrospective study was as conducted with 764 Caucasian older adults with severe obesity (age range: 60-74 years and BMI ≥ 35 kg/m/²). Four models were used to test the accuracy of anthropometry and body composition variables in multivariable prediction of REE. All models were derived by stepwise multiple regression analysis using a calibration group of 382 subjects [295 females and 87 males] and the equations were cross-validated in the remaining 382 subjects [295 females and 87 males] as validation group. The new prediction equations and the other published equations were tested using the Bland-Altman method. Prediction accuracy was defined as the percentage of subjects whose REE was predicted within ± 10% of measured REE.

**Results:**

All the equations analyzed predicted higher energy requirements for males than females, and most of them underestimated the energy requirement values of our sample. The highest accuracy values were observed in the new equations, with 62% in the anthropometric model and 63% in the body composition model.

**Conclusion:**

Although the accuracy of our equations was slightly higher in comparison with the other taken into consideration, they cannot be considered completely satisfactory for predicting REE in Italians older adults with severe obesity. When predicting equations cannot guarantee precise or acceptable values of REE, the use of indirect calorimetry (if available) should be always recommended, especially in clinical practice.

## 1 Introduction

The aging population is increasing worldwide and, in parallel, it is also observed an increase in the prevalence of older people with obesity (1), which is caused by a chronic energy imbalance wherein energy intake regularly exceeds energy expenditure (2).

An important factor to be considered in older adults with obesity is the pronounced body composition changes, with greater deposition of fat mass in visceral organs, at the expense of the amount of fat-free mass, particularly muscle mass (3). These age-related changes are due to the interplay between aging, inflammation, bad eating habits, metabolic disorders, and oxidative stress (4).

It is well established that both aging and obesity, contribute to altering the Resting Energy Expenditure (REE), which represents in most individuals around 50-70% of the total daily energy expenditure (5), which also includes energy consumed during food thermogenesis and physical activity. REE is considered an important parameter to provide a proper caloric intake essential to maintain the ideal energy balance and/or to determine weight loss in subjects with obesity (6).

The most common method of measuring REE in clinical is Indirect Calorimetry (IC) (7) which measures oxygen and carbon dioxide concentrations in the expired air, and then calculates the energy expenditure with equations based on the concentration values of these gases (8). Although the IC is a non-invasive and reliable method, its application is limited because it requires specialized equipment and trained personnel, which can be costly in daily clinical practice (9).

Thus, several predictive equations developed to calculate the REE have already been created in the last 10 years, and a large number of studies proposed new equations and/or validated the existing ones. However, they included data analysis considering adults with obesity without age differentiation (10–13), normal weight and/or older adults (10, 14–16). Other equations were proposed considering groups of people classified by gender (17–19), ethnicity (20, 21), hospitalization (22) and the presence of chronic diseases (23). Despite being widely referenced, these equations were designed for the general population, without considering existing divergences related to age groups and other epidemiological and body composition variables.

In a recent systematic review, Cioffi et al. (24) evaluated the prediction accuracy of REE in healthy older adults according to the group level, and after analyzing 14 included studies, they concluded that, to date, no equation could be recommended because of the criteria limitation. To the best of our knowledge, to date, two equations for older adults with obesity have been validated. The study performed by Noreik et al. (5) found the best accuracy for estimating REE in the equation proposed by Lührmann et al. (3) (0.876, p = 0.0001), which however was elaborated only with body weight and did not take into account body composition variables. In another recent validation study performed by Griffith et al. (6), the best accuracy (59%) for older adults with obesity was observed in the equation proposed by FAO/WHO/ONU (1985), which however was not considered completely satisfactory.

Given the above findings, it remains important to investigate new accurate predictive equations to determine the REE in older adults with obesity, considering their differences according to subgroups (age, gender, ethnicity, and body composition), since these results could help to improve the health strategies in this population at-risk. Thus, this study aimed to develop and validate new predictive equations for REE, based on data from indirect calorimetry, in Italian older adults with severe obesity.

## 2 Subjects and methods

### 2.1 Study sample and design

A retrospective study was performed on 764 Caucasian older adults with severe obesity (590 females and 174 males) hospitalized between January 2016 and January 2019 at the Division of Metabolic Diseases, Italian Institute for Auxology, IRCCS, Piancavallo (VB), Italy, for a 3-week multidisciplinary integrated body weight reduction program (BWRP). Briefly, the BWRP entailed an energy-restricted diet in combination with physical rehabilitation (moderate aerobic activity), psychological counseling, and nutritional education (25).

The inclusion criteria were: 1) age between 65 and 74 years; and 2) Body Mass Index (BMI) calculated as weight (kg)/height² (m) ≥ 35 kg/m^2^, according to the cut-off for obesity in older people recommended by the Pan American Health Organization (2001). Individuals who had overt metabolic and/or endocrine diseases (i.e. diabetes, hypothyroidism, hypertension, amenorrhea), acute or chronic kidney/liver diseases, acute or chronic infection or inflammatory conditions, autoimmune diseases, malignant diseases, neurodegenerative diseases, hematological and/or oncological disorders, and those taking medications regularly or using any drugs known to influence energy metabolism were excluded. For each participant, anthropometric and instrumental measurements, such as the evaluation of body composition by bioimpedance analysis (see below for details), were collected.

This study was approved by the Ethical Committee of Istituto Auxologico Italiano, IRCCS, Milan Italy (code: 2023_03_21_06; research code: 01C311, acronym: EQUASTIMET60). Written informed consent was signed by all participants at the admission to the Hospital.

### 2.2 Anthropometric measurements and body composition

All the anthropometric measurements, REE, and body composition analysis were taken before the beginning of the BWRP by the same trained operators, according to the Anthropometric Standardization Manual (26).

The body weight (BW) was measured to the nearest 0.1 kg using an electronic scale (Ro WU 150, Wunder Sa.bi., Trezzo sull’Adda, Italy). Standardizing height was determined by a Harpenden Stadiometer (Holtain Limited, Crymych, Dyfed, UK).

Body composition was measured by using a multifrequency tetrapolar impedancemeter (BIA, Human-IM Scan, DS-Medigroup, Milan, Italy) with a delivered current of 800 A at a frequency of 50 kHz. To reduce errors of measurement, attention was paid to the standardization of the variables that affect measurement validity, reproducibility, and precision. Measurements were performed according to the method of Lukaski et al. (27) after 20 minutes of rest in a supine position with relaxed arms and legs without any contact with other body parts. Fat-free mass (FFM) was estimated using the prediction equation for adults developed by Bedogni et al. (28). Fat Mass (FM) was obtained by subtracting FFM from body weight (BW) and % FM as (FM/BW) × 100.

### 2.3 Resting energy expenditure

REE was determined in a 22 to 25°C room in the morning (between 08:00 and 10:00 a.m.) and after an overnight fast using an open-circuit, indirect computerized calorimetry with a rigid, transparent, ventilated canopy (Vmax 29, Sensor Medics, Yorba Linda, CA), periodically undergone to quality control tests to ensure the reliability of the measurements. Before each test, the gas analyzers were calibrated using a reference gas mixture (15.00% O_2_ and 5.00% CO_2_). The subjects were fasting from at least 8 hours, were not smoking for at least 1 hour, and waited 30 minutes in a sitting position before undergoing REE measurement. After achieving a steady state in the lying position, the REE of individuals was measured for 30 minutes. The data relative to the acclimation period were discarded. The steady state was defined as at least 5 minutes< 5% variation in the respiratory quotient,< 10% variation in O_2,_ and< 10% variation in minute ventilation (29). Oxygen consumption and carbon dioxide production, standardized for temperature, barometric pressure and, humidity, were recorded at 1-min intervals for a minimum of 30 min and averaged over the whole measurement period. Energy expenditure was derived from the measured oxygen uptake and carbon dioxide output according to the equation of Weir (30).

### 2.4 Statistical analysis

Values of continuous variables are given as mean and standard deviation or median and interquartile range (IQR), according to the data distribution. The data were analyzed using the Kolmogorov–Smirnov test to verify the normal distribution. Student’s t-test or Mann–Whitney U test was used to compare continuous variables between groups, as appropriate. For the development and cross-validation of new equations for predicting REE, the data set of 764 measured REE values was randomly split into two groups: a calibration group (n: 382, 295 females and 87 males) for the development of predictive equations, and a cross-validation group (n: 382, 295 females and 87 males) for the validation of the predictive equations. In the calibration group, the univariable relationships between REE and continuous predictors (age, body weight, height, FFM, and FM) were first studied using scatterplots and non-parametric regression plots. A first-degree linear model was as accurate as more complex models to describe all the REE-predictor relationships and was thus chosen as the reference model for all univariable analyses. Four pre-specified models were used to test the accuracy of anthropometry and body composition in multivariable prediction of REE. Model 1 was based on body weight, age, and gender; Model 2 added height to the predictors of Model 1; Model 3 was based on age, gender and FFM; Model 4 added FM to the predictors of Model 3. Standard diagnostic plots were used to test univariable and multivariable model fit. The adjusted coefficient of determination (R^2^_adj_) and the root mean squared error of the estimate (RMSE) were used as measures of model fit.

The new prediction equations and the other published equations were tested in the cross-validation group by using the Bland-Altman method (31) to ascertain the degrees of systematic and magnitude of bias when predicted REE was compared with measured REE. In addition, prediction accuracy was defined as the percentage of subjects whose REE was predicted to be within ±10% of measured REE. This error limit on prediction accuracy was accepted empirically as being consistent with calorimetry measurement errors of 5% or less (32). Differences between predicted REE and measured REE higher than ±10% were considered prediction errors and are reported as percentages of subjects whose REE was overestimated or underestimated.

Statistical analysis was performed using STATA 18.0 (STATA Corp, College Station, TX, USA).

## 3 Results

The main characteristics of 764 older adults with severe obesity (590 females and 174 males) are shown in **Table 1**. Female and male subjects with obesity had a comparable age between 65 and 74 years and showed a mean BMI of 40.3 kg/m2 [37.4 - 44.8] for females and 38.5 kg/m2 [35.5 - 41.5] for males (p-value< 0.001). Male subjects with obesity were significantly taller (+8.9%, p-value< 0.001) and had the highest FFM values compared to females (+39.5%, p-value< 0.001), while female subjects with obesity showed a higher amount of FM (+19.7%, p-value< 0.001). The REE obtained with indirect computerized calorimetry was significantly higher in males (+20.8%, p-value< 0.001) compared to female patients, but REE normalized for FFM was significantly lower in males than females (-13.7%, p-value< 0.001).

**Table 1:** Physical characteristics of the sample (n:764).

	Females (n=590)	Males (n=174)	p-value
Age (years)	68 (66-70)	68 (66-70)	0.215
Height (m)	1.56 (1.52-1.60)	1.70 (1.65-1.73)	<0.001
Body weight (kg)	98.4 (89.5-110.7)	109.2 (101.5-119.7)	<0.001
BMI (kg/m^2^)	40.3 (37.4-44.8)	38.5 (35.5-41.5)	<0.001
FFM (kg)	48.8 (44.1-54.2)	68.1 (62.8-74.6)	<0.001
FM (kg)	49.8 (45.4-56.0)	41.6 (38.6-46.1)	<0.001
REE (kcal)	1607.5 (1466.9-1765)	1941.5 (1755-2225)	<0.001
REE/FFM (kcal/kg)	32.8 (29.9-35.8)	28.3 (25.9-31.9)	<0.001

All results are median (IQR)

BMI, Body Mass Index; FFM, Fat-Free Mass; FM, Fat Mass; REE, Resting Energy Expenditure

p-value for independent Student t-test for comparison between gender.

The association between the obtained REE and the physical variables was analyzed through univariate regression analysis (**Table 2**). A significant association was observed between the REE and all the tested variables: male gender, age, height, BW, BMI, FFM and FM.

**Table 2:** Univariable regression analysis between anthropometric and body composition variables and Resting Energy Expenditure (REE) in the calibration group (n: 382, 295 females and 87 males).

REE (kcal)	β	95% CI	p-value	R^2^_adj_
Male	354.1	285.1,423.2	<0.001	0.2089
Age (years)	-12.6	-25.1,-0.05	0.049	0.0075
Height (m)	1564.7	1219.8,1909.5	<0.001	0.1710
Body weight (kg)	11.7	10.0,13.3	<0.001	0.3431
BMI (kg/m^2^)	16.8	11.7,21.8	<0.001	0.0988
FFM (kg)	19.2	17.1,21.4	<0.001	0.4490
FM (kg)	8.7	5.0,12.5	<0001	0.0513

BMI, Body Mass Index; FFM, Fat-Free Mass; FM, Fat Mass; REE, Resting Energy Expenditure.

**Table 3** shows that all models of multivariable regression analysis had similar accuracy for predicting REE in the analyzed sample (R^2^adj ~ 0.46; p< 0.001). The insertion of the height variable (Model 2) did not significantly modify the model of simple anthropometric measurements based on BW, age and gender (Model 1). Similarly, the insertion of the FM variable (Model 4) did not substantially change the fit value of the model for body composition based on age, gender and FFM (Model 3).

**Table 3:** Multivariable regression analysis between anthropometric and body composition variables and Resting Energy Expenditure (REE) in the calibration group (n: 382, 295 females and 87 males).

		Model 1	Model 2	Model 3	Model 4
Body weight (kg)		9.9** (8.4,11.4)	10.0** (8.4,11.6)	–	–
Age (years)		-10.2* (-19.5,-0.87)	-10.2* (-19.5,-0.8)	-11.4* (-20.7,-2.07)	-10.7* (-20.2,-1.3)
Male		264.4** (205.2,323.6)	270.4** (196.2,344.7)	10.5 (-68.0,89.0)	144.2 (-163.3,451.8)
Height (cm)		–	-52.4 (-438.6,333.7)	–	–
FFM (kg)		–	–	18.9** (16.0,21.8)	14.2* (3.3,25.1)
FM (kg)		–	–	–	5.3 (-6.5,17.0)
Intercept		1333.1** (665.7,2000.4)	1406.1** (548.4,2263.8)	1474.0** (812.9,2135.1)	1393.9** (708.9,2078.8)
RMSE		239.1	239.4	239.1	239.2
R^2^_adj_		0.4546	0.4533	0.4544	0.4541
P model		<0.001	<0.001	<0.001	<0.001

FFM: Fat-Free Mass; FM: Fat Mass; RMSE: Root Mean Squared Error. Model 1: body weight, age and gender; Model 2: body weight, age, gender and height; Model 3: age, gender and FFM; Model 4: age, gender, FFM and FM. *p<0.05 **p<0.001

Therefore, the new equations for the prediction of REE in older adults with severe obesity are the following:

REE (kcal)= 9.9*BW – 10.2*Age + 264.4*Gender + 1333.1 [anthropometric model 1];

REE (kcal)= –11.4*Age +10.5*Gender +18.9*FFM+ 1474.0 [body composition model 3];

where gender = 1 for males and 0 for females, age in years, BW and FFM in kg.

All equations, with both anthropometric and body composition measurements, predicted higher energy requirements for males than females (p<0.001) (**Table 4**).

**Table 4:** Resting Energy Expenditure (REE) measured by calorimetry and estimated by predictive equations in the validation group (n: 382).

	All (n: 382)	Females (n: 295)	Males (n: 87)	p
Measured REE_calorimetry (kcal)	1707 ± 308	1623 ± 248	1991 ± 321	0.001
Predictive equations based on anthropometric measurements				
New equation (kcal/d)	1717 ± 217	1629 ± 149	2013 ± 137	0.001
Harris-Benedict (kcal/d) (1918/1919)	1675 ± 234	1582 ± 146	1990 ± 198	0.001
Mifflin-St Jeor (kcal/d) (1990)	1561 ± 223	1479 ± 166	1842 ± 152	0.001
FAO/WHO/UNU (kcal/d) (1985)	1727 ± 220	1646 ± 154	2002 ± 182	0.001
Pavlidou (kcal/d) (2023)	1660 ± 210	1571 ± 128	1960 ± 146	0.001
Schofield (kcal/d) (1985)	1643 ± 198	1567 ± 133	1902 ± 158	0.001
Luhrmann (kcal/d) (2002)	1777 ± 220	1704 ± 176	2027 ± 162	0.001
Fredrix (kcal/d) (1990)	1798 ± 210	1723 ± 160	2055 ± 147	0.001
Predictive equations based on body composition measurements				
New equation (kcal/d)	1719 ± 219	1630 ± 140	2021 ± 160	0.001
Cunningham (kcal/d) (1980)	1685 ± 249	1584 ± 158	2029 ± 184	0.001
Mifflin-St Jeor (kcal/d) (1990)	1474 ± 223	1384 ± 142	1782 ± 165	0.001
Korth (kcal/d) (2007)	1693 ± 284	1578 ± 180	2086 ± 210	0.001
Owen (kcal/d) (1988)	1458 ± 267	1349 ± 170	1826 ± 198	0.001
Weigle (kcal/d) (1988)	1344 ± 231	1250 ± 147	1663 ± 171	0.001
Batista (kcal/d) (2023)	1856 ± 454	1671 ± 288	2483 ± 336	0.001

All results are mean ± SD

p-value for independent Student t-test for comparison between genders.

Comparing the REE estimated by indirect calorimetry and the prediction equations analyzed based on anthropometric measurements, it was found that only the new equation proposed and the equation proposed by FAO/WHO/UNU (33) did not show a significant difference (**Table 5**). All other equations were significantly different, with most of them underestimating the energy requirement. Significant overestimations were observed in the equations proposed by Luhrmann et al. (3) and Fredrix et al. (37).

**Table 5:** Accuracy of predictive equations for resting energy estimation compared to Resting Energy Expenditure by indirect calorimetry in the validation group (n: 382).

Method for energy estimation	Absolute bias (kcal)	Relative bias (%)	p-value	RMSE	Under<90%	Accurate 90-110%	Over>110%
Predictive equations based on anthropometric measurements							
New equation	10 ± 221	0.6	0.379	221	18	62	20
Harris-Benedict et al. (34)	-32 ± 219	-1.9	0.001	221	22	60	18
Mifflin-St Jeor et al. (35)	-145 ± 221	-8.5	0.001	264	39	54	7
FAO/WHO/UNU (33)	20 ± 221	1.2	0.073	222	16	55	29
Pavlidou (15)	-47 ± 221	-2.8	0.001	226	24	59	17
Schofield (36)	-64 ± 221	-3.7	0.001	230	25	60	15
Luhrmann et al. (3)	71 ± 223	4.1	0.001	234	10	53	37
Fredrix et al. (37)	92 ± 221	5.4	0.001	239	9	51	40
Predictive equations based on body composition measurements							
New equation	12 ± 221	0.7	0.280	221	16	63	21
Cunningham (38)	-21 ± 225	-1.2	0.065	226	23	57	21
Mifflin-St Jeor et al. (35)	-232 ± 222	-13.6	0.001	321	60	37	3
Korth (39)	-13 ± 233	-0.8	0.263	233	22	55	23
Owen (40)	-249 ± 229	-14.6	0.001	338	63	35	3
Weigle (41)	-363 ± 223	-21.2	0.001	425	85	14	1
Batista (14)	149 ± 328	8.7	0.001	360	14	40	46

p-value: Paired t-test comparing predictive equations with the reference method (indirect calorimetry).

RMSE, Root mean square error.

Considering prediction equations based on body composition measurements, the new equation and equations proposed by Cunningham (38) and Korth et al. (39) did not show a significant difference from REE. However, the equations proposed by Mifflin et al. (35), Owen (1988), Weigle et al. (41) and Batista et al. (14) showed a significant underestimation of REE (**Table 5**).

The new proposed equations had accuracies of 62% and 63%, considering anthropometric and body composition models, respectively. Considering the other equations analyzed, the most accurate was the equation proposed by Harris-Benedict (34) and Schofield (36) with 60%, followed by Pavlidou et al. (15) with 59% (**Table 5**).

**Figure 1:**
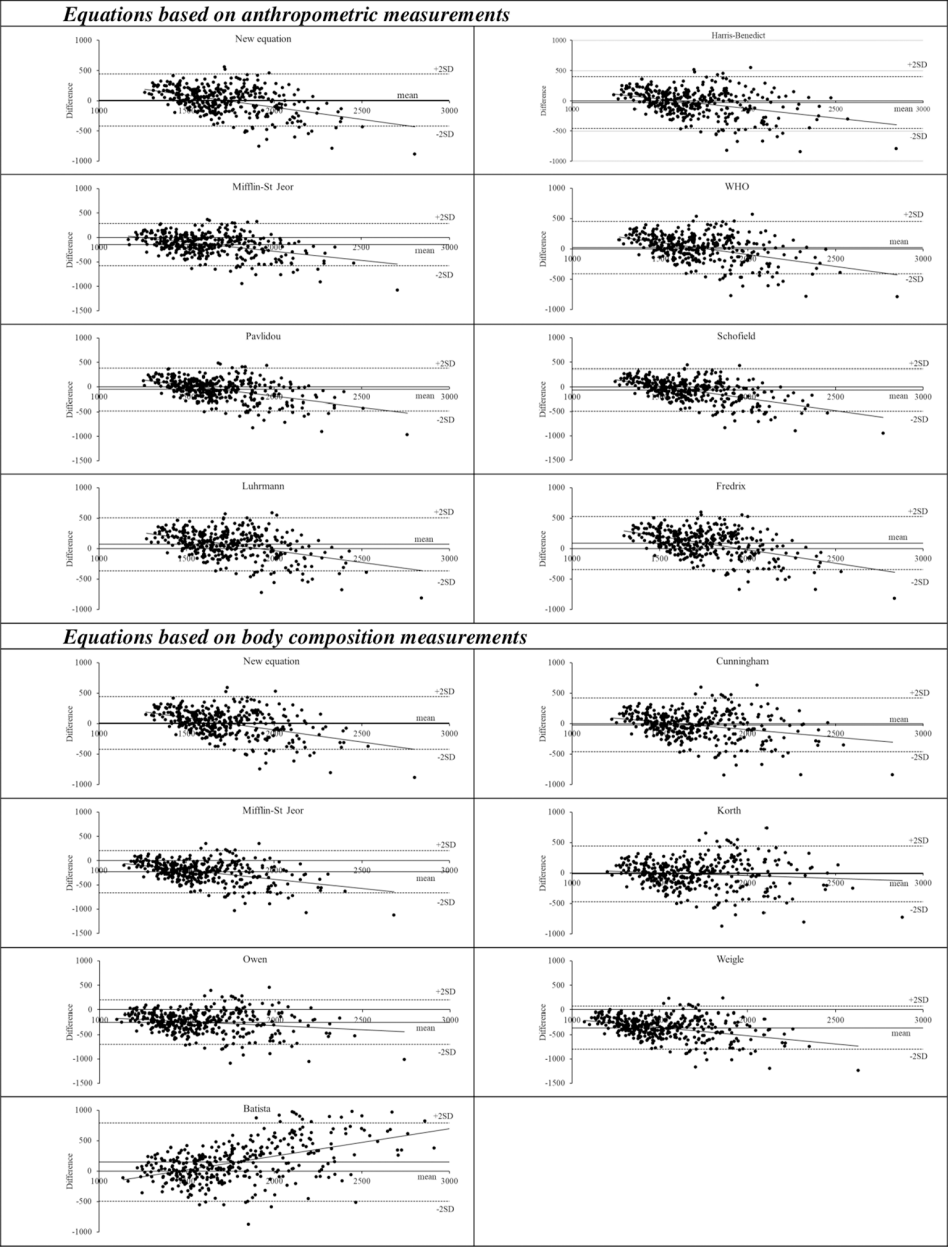
Bland-Altman plots of energy expenditure measured by indirect calorimetry and predicted by fifteen tested equations. The dashed lines represent the mean difference between Resting Energy Expenditure (REE) predicted by each of the tested equations and REE measured by indirect calorimetry and ±1.96 standard deviations (SD) of the mean (limits of agreement), with a 95% confidence interval (dotted lines).

Most of the analyzed equations presented a systematic bias for underestimation of energy requirements, as observed in **Figure 1**. The smallest values of relative bias were found in the new equation, in the FAO/WHO/UNU (33) (1.2%) and in the Harris-Benedict (34) (-1.9%) considering anthropometric models, and in the Korth et al. (39) (-0.8%) and the Cunningham (38) (-1.2%) considering body composition models.

## 4 Discussion

In the present study we proposed new equations to estimate REE in older adults with severe obesity based on data of indirect calorimetry and also, we validated them and other main equations already proposed in the literature. In summary, there was a variation in the previously proposed equations to predict REE in our sample, in which most of them underestimated the energy requirement and had poor accuracy. The best accuracies were found in the new proposed equations, with 62% and 63%, respectively, considering the anthropometric and body composition in the models.

Our results showed that only the new equations and the equation proposed by FAO/WHO/UNU (33) (for anthropometric models) and by Cunningham (38) and Korth et al. (39) (for body composition models) did not show a significant difference between the estimated values ​​and those obtained by using the indirect calorimetry of REE. This evidence contradicts that found by Noreik et al. (5) in which a better accuracy for REE estimation in older adults with obesity was observed in the equation proposed by Lührmann et al. (3). However, this equation was tested in a sample of not hospitalized patients with a mean BMI value of 26 kg/m², which is far lower than the one considered for our sample of severely obese. Moreover, other studies that analyzed samples of old people (3, 14, 15, 36, 37) also included non-obese individuals, making extremely difficult the comparison of the results.

It is important to highlight that advanced aging alters the body composition, with an increase in adipose tissue and a decrease in lean tissues (42), and changes in both the quantity and quality of lean mass which are key factors to determine the REE precision of prediction equations. Due to their greater body mass, individuals with severe obesity require a higher REE than eutrophic people, to maintain their essential body functions (17). However, the weight excess in individuals with obesity has a significant impact on the age-related declines in terms of REE, with a mean decline almost 1.5 times greater than normal weight individuals (43).

Recently, Griffith et al. (6) validated four equations commonly used to guide weight loss interventions in a sample of older adults with obesity (≥ 65 years and BMI ≥ 30kg/m²) and found that FAO/WHO/UNU (33) also had the best accuracy for REE estimation. However, none of them obtained values ​​above 60%. In the present study, although the highest accurate values ​​were observed in the models with anthropometric variables, only the new equation with body composition variables achieved an accuracy of 62%. Other body composition-based prediction models compared in our study had a lower agreement, including the study by Batista et al. (14) with 40%, which was the only one that analyzed a sample of older adults, however non-obese. This finding is in agreement with other studies where FFM values were more pertinent than isolated anthropometric variables during energy requirements estimations (38, 39, 41).

In the present study, the REE obtained by indirect calorimetry was higher in men than in women, and in the same way, all equations predicted higher energy requirements for men. However, when REE values were normalized to the FFM, higher REE values were observed ​​in women. The greater amount of fat mass in older women than in men could lead to a lower basal metabolic rate and, consequently, lower energy (18), thus reinforcing the importance of considering FFM values during REE estimations. Also, it is important to note that the inclusion of the FM variable in our prediction model did not substantially change the predictive estimation of REE calculated only with the FFM. Although our model explained less than 50% of the variance, it is in agreement with other studies that found FFM as the major factor related to REE (38, 44).

Most of the body composition equations tested in our study presented a tendency to underestimate the REE values. As previously mentioned, body composition, especially the FFM values of the sample and other measures of nutritional status are the main factors that influence the REE. In addition to the differences in clinical characteristics of the samples, the methods used in each study to calculate body composition should be considered to explain these variations. While we used BIA measurements, other tested equations were created based on skinfold measurements (38, 40) or densitometry methods (i.e. DXA) (14). It is also important to mention that other variables not included in our analysis may contribute to the variations observed in the REE of older adults with severe obesity. According to Martin et al. (45), the race can significantly affect REE variability. In fact, these authors demonstrated that black individuals typically have lower REE than white participants. Another anthropometric characteristic described in the literature is waist circumference, which has been shown to tend to increase in women with aging (46).

Some limitations of our study should be considered while interpreting the results. The new equations might not be entirely representative of European older adults with obesity being the average BMI of our sample considered representative of a severe obesity condition. Also, the sample was recruited in a hospital setting, and patients with serious illnesses were excluded. Therefore, body composition variables were measured by BIA, while DXA is still considered the gold standard method for the estimation of fat mass and fat-free mass.

Nevertheless, REE was measured by indirect calorimetry with a highly accurate method. To the best of our knowledge, our study was the first to propose specific equations for European older adults with severe obesity, with a mean BMI of 40.3 kg/m² for women and 38.5 kg/m² for men. Furthermore, our equation based on body composition measurements achieved an accuracy greater than 60%, which had not been found in previous studies. The validation analysis considered all the main equations used in the last decades, highlighting the importance of using specific equations for each population.

In conclusion, although the accuracy of our equations was slightly higher (62% and 63% considering anthropometric and body composition models, respectively) in comparison with the other available equations taken into consideration, they cannot be considered completely satisfactory for predicting REE in Italians older adults with severe obesity. To date, we cannot exclude that the use of these equations in other different populations might provide more accurate results than those obtained in the Italian population from which formulas have been developed. When predicting equations cannot guarantee precise or acceptable values of REE, the use of indirect calorimetry (if available) should be always recommended, especially in clinical practice.

## Data availability statement

The raw data supporting the conclusions of this article will be uploaded on Zenodo.org immediately after the publication of the manuscript and they will be available upon a reasonable request to the corresponding author.

## Ethics statement

The studies involving humans were approved by Ethical Committee of Istituto Auxologico Italiano, IRCCS, Milan Italy (code: 2023_03_21_06; research code: 01C311, acronym: EQUASTIMET60). The studies were conducted in accordance with the local legislation and institutional requirements. The participants provided their written informed consent to participate in this study.

## Author contributions

AD: Conceptualization, Investigation, Methodology, Resources, Validation, Visualization, Writing – original draft, Writing – review & editing. SL: Conceptualization, Data curation, Formal Analysis, Investigation, Methodology, Project administration, Software, Supervision, Validation, Writing – original draft, Writing – review & editing, Resources, Visualization. AM: Data curation, Methodology, Validation, Visualization, Writing – original draft, Writing – review & editing. LA: Data curation, Validation, Visualization, Writing – review & editing. MD: Data curation, Formal Analysis, Validation, Visualization, Writing – review & editing. MDM: Data curation, Formal Analysis, Validation, Visualization, Writing – review & editing. MI: Data curation, Formal Analysis, Validation, Visualization, Writing – review & editing. NP: Validation, Visualization, Writing – review & editing. VM: Writing – review & editing. AL: Visualization, Writing – review & editing. AS: Conceptualization, Data curation, Funding acquisition, Investigation, Methodology, Project administration, Resources, Supervision, Validation, Visualization, Writing – review & editing, Writing – original draft.
